# Post-SARS-CoV-2 Acute Telogen Effluvium: An Expected Complication

**DOI:** 10.3390/jcm11051234

**Published:** 2022-02-24

**Authors:** Paola Monari, Giulio Gualdi, Giorgio Bettoni, Raffaella Costa, Giorgio Ragni, Francesca Zani, Giovanna Bianchi, Silvia Casella, Elisa Casella, Massimo Crippa, Piergiacomo Calzavara Pinton, Marta Di Nicola, Annamaria Porreca, Paolo Amerio, Pierangelo Guizzi

**Affiliations:** 1 Department of Dermatology, ASST Spedali Civili Brescia, 25123 Brescia, Italy; paola.monari@libero.it (P.M.); piergiacomo.calzavarapinton@unibs.it (P.C.P.); 2 Dermatologic Clinic, Department of Medicine and Aging Science, Università G. D’Annunzio Chieti-Pescara, 66100 Chieti, Italy; 3 Internal Medicine Department, Ospedale di Gardone Val Trompia, 25063 Brescia, Italy; giorgio.bettoni@asst-spedalicivili.it (G.B.); raffaella.costa@asst-spedalicivili.it (R.C.); giorgio.ragni@asst-spedalicivili.it (G.R.); francesca.zani@asst-spedalicivili.it (F.Z.); giovanna.bianchi@asst-spedalicivili.it (G.B.); silvia.casella@asst-spedalicivili.it (S.C.); elisa.casella@asst-spedalicivili.it (E.C.); massimo.crippa@asst-spedalicivili.it (M.C.); 4 Biostatistic Laboratory, Department of Experimental and Clinical Sciences, G. D’Annunzio University, 66100 Chieti, Italy; marta.dinicola@unich.it (M.D.N.); annamaria.porreca@unich.it (A.P.); 5 Department of Orthopedic Surgery, Ospedale di Gardone Val Trompia, 25063 Brescia, Italy; pierangelo.guizzi@asst-spedalicivili.it

**Keywords:** SARS-CoV-2, COVID-19, telogen effluvium, alopecia

## Abstract

Post-SARS-CoV-2 telogen effluvium has been described in case reports of COVID-19 patients. We evaluated the prevalence of post-SARS-CoV-2 telogen effluvium in patients from a single medical center, exploring any causal links with the infection. Our hospital-based, cross-sectional study was conducted with patient participants discharged with a diagnosis of SARS-CoV-2 pneumonia from 1 March to 4 April 2020. All patients were evaluated by the same senior dermatologist; a clinical/dermatoscopic evaluation was performed. Alopecia was assessed in 31.3% of patients, with a significant difference in sex (females 73%, males 26.7%). The average time detected from the onset of the first symptoms to alopecia was 68.43 days. Overall, there were no significant associations between alopecia and COVID-19-related features (length of hospitalization, virologic positivity, or duration of fever), treatment characteristics, or laboratory findings. In this paper, we report that post-infection acute telogen effluvium occurs in a significant number of COVID-19 patients. The burden of this condition may impair the quality of life, with a significant impact on individuals.

## 1. Introduction

In December 2019, an outbreak of coronavirus disease (COVID-19)—caused by severe acute respiratory syndrome coronavirus 2 (SARS-CoV-2), a positive-sense single-stranded RNA virus—was reported as a public health emergency of international concern [1]. COVID-19 rapidly spread from China to the entire world, affecting from January to June 2020, over 22 million people across 215 countries [2]. No specific treatment is yet available for COVID-19, and patient management relies on supportive care [3]. Approximately 31 to 41.8% of hospitalized COVID-19 patients rapidly develop acute respiratory distress syndrome (ARDS), with an increased risk of death [4,5]. Patient deterioration is likely related to a dysregulated systemic inflammation [6], due to the increase in the serum levels of inflammatory cytokines [7]. Patients with ARDS are admitted to Intensive Care Units (ICU) with severe hypoxemia, extrapulmonary organ failures, and a marked inflammatory response. Survivors lose weight and are debilitated, often with cognitive impairments. Metabolic control is often disrupted, and internal organs undergo microscopic damages during acute inflammation [8]. Telogen effluvium is a scalp disorder characterized by diffuse, non-scarring hair shedding [9]. The term “telogen effluvium” (TE) was proposed to differentiate the disorder from the excessive shedding of normal club hair [10]. It affects both males and females, with a higher incidence rate in females [9]. Various hypotheses have been proposed for the pathophysiology of TE, based on abnormalities in the normal hair cycle, triggered by different factors. Headington et al. [11] suggested the existence of five different functional types of telogen effluvium, based on alternations in particular phases of the follicular cycle [10]. Most of the cases of TE are subclinical; therefore, its true incidence is not precisely known [11]. Acute TE (ATE), defined as hair shedding lasting for less than six months, can be taken into consideration when hair shedding exceeds 100 hairs every 5 days [11]. Generally, hair loss occurs two to three months after exposure to a trigger or an underlying condition. However, in around 33% of cases, the cause remains unknown [12]. ATE usually undergoes remission in around 95% of cases. Usually, the resolution of the effluvium is associated with the appearance of shorter, re-growing frontal hair which can easily be observed using video dermoscopy [9,13,14]. The most indicative dermoscopic clue of telogen effluvium is the lack of features typical of other diseases [15,16,17]; common but non-specific findings include the presence of empty hair follicles, a predominance of follicular units with only one hair, perifollicular discoloration (the peripilar sign), upright regrowing hairs (mainly acute forms) and progressive uniform hair thinning (chronic forms) [15,16,17]. On trichoscopy, telogen effluvium is a diagnosis of exclusion, whereas the histopathology of the acute forms is non-specific, and resembles that of a normal scalp [10].

During the epidemic, we observed an abnormally high frequency of increased scalp hair shedding in post-infected individuals, without scarring or permanent hair loss. This type of hair loss occurred early after subject-intensive care (SIC) hospitalization. The SARS-CoV-2 infection showed an increase in proinflammatory cytokines which can initiate the process leading to TE by damaging the matrix cells [10]. In particular, the high levels of interferons have already been confirmed to be associated with ATE [18]. At the same time, the multi-drug regime of COVID-19 treatment had also been proposed as an alternative explanation for COVID-associated ATE [19].

Numerous single case reports or case series have been published, however, none were based on prospective cohort studies assessing the true incidence of the disease.

Thus, we decided to study the incidence of TE following COVID-19 infection.

## 2. Materials and Methods

A hospital-based, cross-sectional study was conducted, enrolling participants among patients discharged with a diagnosis of SARS-CoV-2 pneumonia from 1 March to 4 April 2020.

The study population consists of consecutive adult SARS-CoV-2 patients (pneumonia with RT-PCR positive for SARS-CoV-2) admitted to SIC. In the course of the study, informed written consent was obtained from all participants in accordance with the Declaration of Helsinki.

### 2.1. Alopecia Assessment

All patients were monthly evaluated by the same senior dermatologist. The onset of TE was calculated starting from the date of discharge. A clinical and dermoscopic evaluation was performed at each visit, which allowed to differentiate two groups: “cases” vs. “non-cases”. A previous history of alopecia and/or hair problems was also obtained.

### 2.2. Clinical Covariate Assessment

Routine demographic information was collected from participants. Other information was obtained from patient records and included: dates of beginning and end of COVID-19-related fever, swab positivity, inpatient admission and discharge, pharmacological treatment, and oxygen supplementation.

### 2.3. Laboratory

Data from laboratory tests were also collected, including complete blood count (CBC), blood iron, ferritin, transferrin, and zinc concentration, as well as triiodothyronine and thyroxine blood concentrations. All laboratory tests were performed during hospitalization and 2 months after discharge.

### 2.4. Statistics

Continuous variables were presented as mean (±SD), and categorical variables were written as numbers (%). We compared proportions for categorical variables between the groups using the Chi-square test. When values were normally distributed, we used independent group t-tests to compare means of continuous variables between the groups; otherwise, we used the Mann–Whitney U test. Analyses were performed using the R software environment for statistical computing (version 3.4.1; http://www.r-project.org/; accessed on 1 November 2020).

## 3. Results

### 3.1. Characteristics of Participants

Ninety-six patients were consecutively enrolled in the study (Table 1). The mean age of the patients was 59.0 years (54.5–65.0) and the majority were males (*n* = 62, 64.6%; females *n* = 34, 35.4%), with no significant difference in mean age. Patients were hospitalized for an average of 13 days (9.0–16.5). The mean duration of COV-2 positivity was 31.0 days [26.0–37.0], while the mean duration of fever was 11.0 days (9.0–13.0). As for treatment, 83 (86.5%) patients received hydroxychloroquine, 24 (25%) steroids, 59 (61.5%) azithromycin, 31 (32.3%) anticoagulants or antiplatelet agents for pulmonary embolism prophylaxis, and 73 (76%) needed oxygen therapy. Ritonavir was the most used antiviral drug (94 patients, 97.9%), followed by darunavir, administered to 84 patients (87.5%), while lopinavir was given to 24 patients (25%).

### 3.2. Post-COVID Alopecia Characteristics

**Figure 1:**
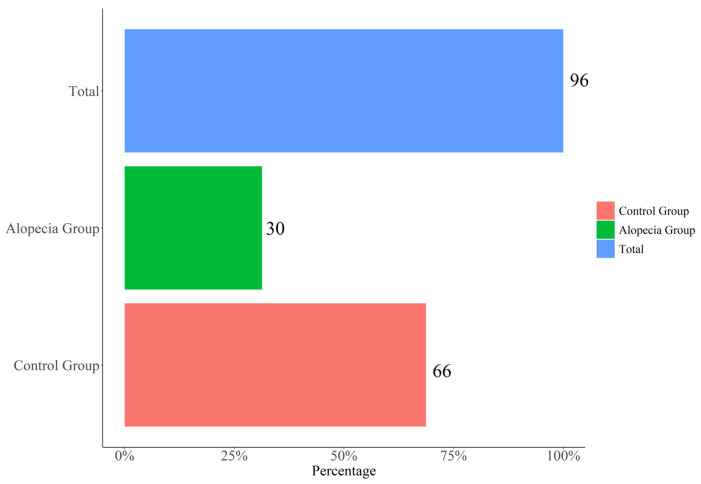
Alopecia frequency in SARS-CoV-2 pneumonia patients.

Alopecia was assessed in 30 of the 96 patients (31.3%) (Figure 1), of whom 22 (73.3%) were females and 8 (26.7%) males, with a significant difference in gender. The average time elapsed from the onset of the first symptom (fever) to that of alopecia was 68.43 days, with a difference between females (72.36) and males (54.00). Eight patients (26.6%) reported trichodynia as the initial symptom of telogen effluvium.

**Figure 2:**
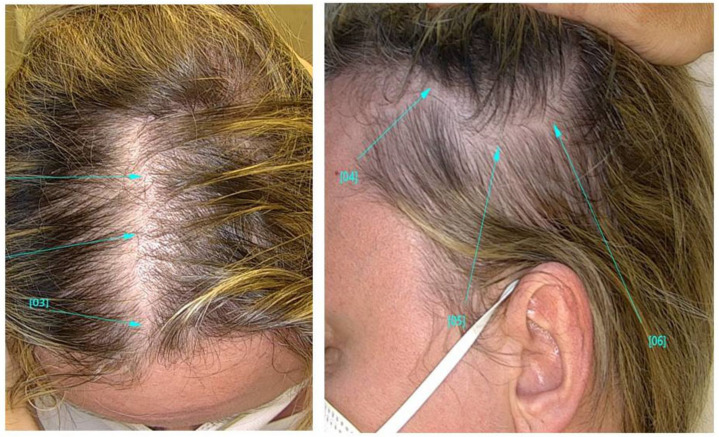
Macroscopic photo showing diffuse alopecia. The arrows indicate typical areas of alopecia.

**Figure 3:**
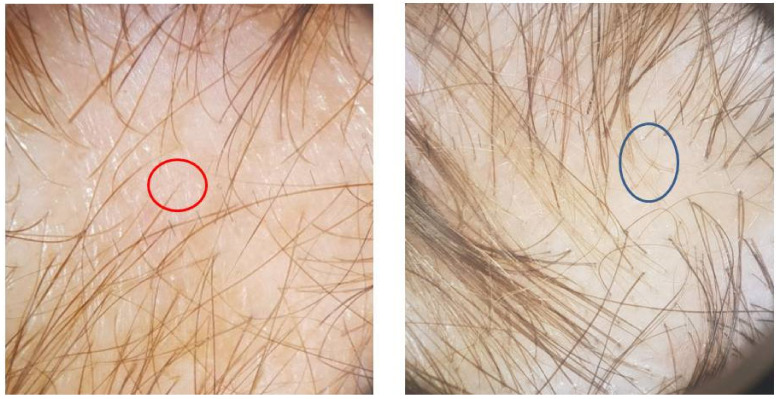
The predominance of single-hair follicular units, with perifollicular discoloration and generalized tiny hair (red circle). Note the presence of upright regrowing hairs (blue circle).

TE involved the whole scalp, with a gradual loss of hair volume in all patients reporting increased hair loss. (Figure 2). Women, in particular, reported difficulty in bending their hair. Dermoscopy (trichoscopy) was useful for the purpose of making a good assessment of the rarefaction and concentration of vellus hair, in order to differentiate TE from androgenic alopecia (Figure 3). Images of the vertex were collected and compared to those of the occipital and the supra-auricular areas. At trichoscopy, most of the hair had a normal appearance. In addition, follicular openings with only one hair predominated. Dermoscopy showed no significant changes between the vertex area, the parietal zone, and the occipital zone, with no miniaturization, and vellus hair allowed to exclude the diagnosis of androgenetic alopecia in all patients.

### 3.3. COVID-19 Characteristics and Alopecia

Overall, there was no association between COVID-19-related characteristics (days of hospitalization, days of COV-2 positivity, days with fever, and TE (Table 1). The disease was not associated with any COVID-directed therapy (Table 1).

### 3.4. Laboratory and Alopecia

Overall, there was no association between the laboratory test outcomes of the control group and the patients with TE. In particular, the levels of iron (*p* = 0.371), ferritin (*p* = 0.194), transferrin (*p* = 0.890), and zinc (*p* = 0.375) were similar in patients with and without TE.

## 4. Discussion

The current study is the first prospective cohort study examining the prevalence of TE following SARS-CoV-2 infection. Previous short reports on a limited number of patients showed an increase in the incidence of post-SARS-CoV-2 TE [20], with particular reference to some ethnic groups [20]. We report a higher incidence of TE in patients after SARS-CoV-2, with about one-third of them presenting with this condition. Due to the TE multifactorial etiology, it’s unclear if this occurrence is more closely related to the stress associated with the fear of the pandemic or, as we think, if it’s triggered directly by the infection. Indeed, it should be considered that the underlying inflammation may be a triggering factor in a subset of patients [21]. Virus infections have been reported in the literature as having a relationship with the onset of TE. This may be due to direct virus aggression of human hair follicle dermal papilla cells as demonstrated for Dengue virus infection in Taiwan in 2014 and 2015 [22]. Other cases have been described in patients with HIV infection, triggered by infectious or associated diseases, nutritional deficiencies, or, often by antiviral drugs [23]

The rate of post-SARS-CoV2 TE appeared to be significantly higher in females, although in males the onset of alopecia was more premature. This occurrence could be linked to the evidence that men infected with CoV-2 have an increased risk of severe COVID-19 disease compared to women [24]. A large number of other factors have been associated with this gender disparity. There is evidence that supports the role of androgens in the COVID-19 severity [25,26], to the extent that androgenic alopecia has been hypothesized as a predicting factor for more severe SARS-CoV-2 cases [27]. In our study, we report TE not as a causative factor, but as the consequence of the disease. Physiological stress, such as childbirth, surgical trauma, high fever, chronic systemic illness or infections, and hemorrhage have been associated with TE. However, the relationship between TE and emotional stress is uncertain and ambiguous [23]. TE has also been linked to severe protein, fatty acid and zinc deficiency, chronic starvation, and caloric restriction [23]. Numerous drugs can cause TE, starting after Week 12 of dosage [11,28]. Iatrogenic causes of TE include oral contraceptive pills, androgens, retinoids, beta-blockers, angiotensin-converting enzyme inhibitors, anticonvulsants, antidepressants, and anticoagulants (heparin) [29]. The relation between TE and anticoagulant therapy is strongly controversial in the literature [19]; however, in our study, no association was manifest. The onset of hair loss in our sample started just over 2 months from the beginning of the fever but, at the same time, it showed no link with any significant laboratory abnormalities. In particular, zinc and iron deficiencies were not related to the onset of alopecia. In the context of these considerations, ATE following SARS-CoV infection is to be considered as a foreseeable complication directly related to the disease. Our report on the absence of any association with the severity of infection and treatments has major implications on the number of patients potentially affected. The absence of a nutritional deficiency, combined with trichodynia, may suggest that the inflammatory condition inherent to COVID-19 may be a principal cause of ATE. Interestingly, the prevalence of trichodynia—a distinctive symptom of TE—is reported in only about 20% of TE patients [30], whereas in our sample, it affected almost one-quarter of subjects.

The onset of this late complication may lead to profound implications, further impairing the quality of life of patients, despite them having recovered from their primary illness. The burden of post-infection ATE will impair the quality of life and may have a significant impact on individuals, employers, and the healthcare systems. 

## Figures and Tables

**Table 1:** General characteristics of patients involved in the study.

Variable	*OVERALLN* = 96	No-Alopecia *n* = 66	Alopecia *n* = 30	*p*-Value
Gender:				<0.001
F	34 (35.4%)	12 (18.2%)	22 (73.3%)	
M	62 (64.6%)	54 (81.8%)	8 (26.7%)	
Age (years)	59.0 (54.5-65.0)	59.0 (55.2;64.8)	59.0 (53.2;66.5)	0.880
Swab positivity(days)	31.0 (26.0-37.0)	31.0 (26.0;37.0)	29.5 (25.2;34.5)	0.274
Hospitalization (days)	13.0 (9.0-16.5)	14.0 (8.25;17.0)	11.5 (10.0;15.0)	0.444
Fever (days)	11.0 (9.0-13.0)	10.5 (8.25;13.0)	11.0 (9.25;14.8)	0.225
DRUG				
Lopinavir:				0.611
No	72 (75.0%)	48 (72.7%)	24 (80.0%)	
Yes	24 (25.0%)	18 (27.3%)	6 (20.0%)	
Darunavir:				0.330
No	12 (12.5%)	10 (15.2%)	2 (6.67%)	
Yes	84 (87.5%)	56 (84.8%)	28 (93.3%)	
Ritonavir:				0.847
No	2 (2.1%)	2 (3.03%)	0 (0.00%)	
Yes	94 (97.9%)	64 (97.0%)	30 (100%)	
Chloroquine:				0.778
No	13 (13.5%)	9 (13.6%)	4 (13.3%)	
Yes	83 (86.5%)	57 (86.4%)	26 (86.7%)	
Azithromycine:				0.977
No	37 (38.5%)	25 (37.9	12 (40.0%)	
Yes	59 (61.5%)	41 (62.1%)	18 (60.0%)	
O2:				0.233
No	23 (24.0%)	13 (19.7%)	10 (33.3%)	
Yes	73 (76.0%)	53 (80.3%)	20 (66.7%)	
Steroids:				0.611
No	72 (75.0%)	48 (72.7%)	24 (80.0%)	
Yes	24 (25.0%)	18 (27.3%)	6 (20.0%)	
Pulmonary Embolism prophylaxis:				0.049
No	65 (67.7%)	40 (60.6%)	25 (83.3%)	
Yes	31 (32.3%)	26 (39.4%)	5 (16.7%)	
